# Two new combinations, lectotypifications and a new name for Costa Rican *Palicourea* s.l.

**DOI:** 10.3897/phytokeys.80.13330

**Published:** 2017-05-19

**Authors:** Andreas Berger

**Affiliations:** 1 Division of Systematic and Evolutionary Botany, Department of Botany and Biodiversity Research, University of Vienna, Rennweg 14, A-1030 Vienna, Austria

**Keywords:** *Palicourea*, *Psychotria*, Rubiaceae, Mesoamerica, taxonomy

## Abstract

Species of the complex and diverse genera *Psychotria* and *Palicourea* are common but little-known elements in many tropical forest ecosystems. DNA-phylogenetic studies and a re-evaluation of morphological characters have recently shown that species of Psychotria subg. Heteropsychotria are nested within *Palicourea* s.l., which was traditionally separated by exhibiting a bird-pollinated (vs. insect-pollinated) pollination syndrome. In order to render both genera monophyletic groups, species of subg. Heteropsychotria need to be transferred to *Palicourea* s.l. For Central American species, most of the necessary combinations have already been made. In the course of ongoing research on the phytochemical characterization of species and clades of Costa Rican *Palicourea* s.l., the nomenclature of Mesoamerican species was revised. As a result, two new combinations and a new name are proposed here: *Palicourea horquetensis* (Dwyer & Hayden) A. C. Berger & C. M. Taylor is based on *Rudgea horquetensis* Dwyer & Hayden, *Palicourea tonduzii* (K. Krause) A. C. Berger is based on *Cephaelis tonduzii* K. Kraus and *Palicourea longiinvolucrata* A. C. Berger replaces *Psychotria hispidula* Standl. In addition, two lectotypes are designated.

## Introduction

Species of the complex and diverse genera *Psychotria* L. (1759: 929) and *Palicourea* Aubl. (1775: 172–175) are prominent but little-known elements in tropical forest ecosystems. Both have long been considered closely related, and *Palicourea* was differentiated from *Psychotria* by characters associated with hummingbird rather than insect pollination. Species of *Palicourea* are typically found in the understory of rainforests and are especially frequent in high elevation habitats where *Psychotria* and other related genera are less speciose (Taylor 1996, 1997).

Both genera were traditionally classified in the tribe Psychotrieae. Recently, however, DNA-phylogenetic studies and a re-evaluation of morphological characters have shown that species of Psychotria subg. Heteropsychotria Steyerm. (1972: 484) are more closely related to *Palicourea*. Consequently, views shifted towards a narrower concept of *Psychotria* and Psychotrieae which peaked in the ongoing segregation of hundreds of species and the establishment of the sister tribe Palicoureeae (Nepokroeff et al. 1999, Razafimandimbison et al. 2014, Robbrecht and Manen 2006).

Species of Psychotria subg. Heteropsychotria and *Palicourea* cannot be distinguished by vegetative or fruit characters. In addition, both groups show similar accumulation of alkaloids (e.g., Berger et al. 2012, 2015, 2017, in review), flavonoids (e.g., Berger et al. 2016) and a group of defensive peptides termed cyclotides (Koehbach et al. 2013). As traditionally defined, both groups deviate only in a suite of traits associated with pollination syndromes: Flowers in subg. Heteropsychotria are arranged in open, somewhat grouped to densely capitate inflorescences with inconspicuously colored inflorescence axes, though some species have inflorescences subtended by showy bracts. Flowers are usually sessile or subsessile and have small, white, to greenish or yellow corollas with short and straight tubes in bee-pollinated or white and long-tubed corollas in moth-pollinated species. (e.g., Steyermark 1972, Taylor 1996).

By contrast, species of *Palicourea* are hummingbird-pollinated, frequently have long-pedunculate and open inflorescences, colored inflorescence axes, large and long pedicellate flowers and vividly colored corollas with well-developed tubes. Corollas have a gibbous, nectar-accumulating swelling at their base that is protected by an internal ring of hairs. In many plant groups, bird-pollinated flowers have repeatedly evolved in groups of bee-pollinated ancestors and are not phylogenetically informative at the generic level (e.g., Castellanos et al. 2004, Fenster et al. 2004, Pirie et al. 2016). Similarly, it was hypothesized that pollinator shift has occurred multiple times in *Palicourea* s.l. and that bird pollinated species (i.e., the traditional concept of *Palicourea*) repeatedly evolved out of bee-pollinated ancestors (i.e., the traditional concept of Psychotria subg. Heteropsychotria) or vice versa (Taylor 1996, 1997).

In order to render both *Palicourea* and *Psychotria* monophyletic groups, most species of Psychotria subg. Heteropsychotria have to be transferred to *Palicourea* s.l. The combined group includes more than 800 species, is variable in flower characters, but is supported by vegetative and fruit characters as well as by DNA phylogenetic data (Razafimandimbison et al. 2014). The process of transferring species of subg. Heteropsychotria was started with the publications of Taylor et al. (2010), Taylor (2015a, 2015b) and Taylor and Hollowell (2016), which provided combinations for species belonging to newly defined sections within *Palicourea* s.l. Finally, Mexican, Mesoamerican and Venezuelan species were transferred by Borhidi (2011, 2017a, 2017b), and species occurring in the Guianas were transferred by Delprete and Kirkbride (2016).

In the course of ongoing research on the phytochemical characterization of species and clades of Costa Rican *Palicourea* s.l., the nomenclature of Mesoamerican species was revised and the need for a new name and two new combinations became apparent. These are proposed here. In addition, a complete synonymy, an enumeration of type specimens and two lectotypifications are provided for these three species.

## Methods

The present work is based on an extensive study of herbarium specimens, digital images and relevant literature including regional (e.g., Manual de Plantas de Costa Rica, Taylor 2014) and overregional floras (e.g., Flora Mesoamericana, Lorence and Taylor 2012). In addition, extensive fieldwork was performed in Costa Rica in 2010, 2013, 2015 and 2016.

For all names, protologues were checked to verify or revise author and page citations, information on collectors and localities. Subsequently, the type category applying to each name was assessed in accordance with the ICN (Melbourne code, McNeill et al. 2012; see also McNeill 2014). Retrieved information was managed with the international JACQ herbarium database (http://herbarium.univie.ac.at/database) hosted at the herbarium WU. For citation of type collections, localities have been simplified and ecological and morphological details have been omitted. For all retrieved type specimens, herbarium acronyms and barcodes are given. For specimens seen either digitally or physically, their barcodes are followed by an exclamation mark.

For the three nomenclatural novelties, both possible author abbreviations of my name are preoccupied: Berger stands for Ernst Friedrich Berger (1814–1853) and A. Berger stands for Alwin Berger (1871–1931). Therefore, I have adopted the abbreviation A. C. Berger that includes my second forename Christoph, which I have never used before in my publications.

## Taxonomy

### Palicourea horquetensis

Taxon classificationPlantaeGentianalesRubiaceae

(Dwyer & Hayden) A. C. Berger & C. M. Taylor comb. nov.

urn:lsid:ipni.org:names:77162933-1

Rudgea horquetensisBasionym. Dwyer & Hayden, Ann. Missouri Bot. Gard. 54(2): 145–146, 1967.—**Type**: PANAMA. Chiriquí: Distr. Boquete, Cerro Horqueta, ca. 1980 m, 26 Jul 1940, *C. von Hagen & W. von Hagen 2156* (holotype: NY barcode 133202!).
Coussarea nebulosa= Dwyer, Ann. Missouri Bot. Gard. 67(1): 131, 1980a. ≡ Psychotria nebulosa (Dwyer) C. M. Taylor, Novon 5(2): 205, 1995, nom. illeg. hom., non Psychotria nebulosa K. Krause, Bot. Jahrb. Syst. 57(1): 46–47, 1920. ≡ Palicourea nebulosa (Dwyer) C. M. Taylor, Novon 20(4): 488, 2010.—**Type**: PANAMA. Chiriquí: Monte Rey near Boquete, ca. 1170 m, 20 Jul 1971, *T. B. Croat 15868* (lectotype, designated by Taylor (1995): PMA barcode 1189! ex MO 2162999 [sheet # 1/2]; isolectotype: MO barcode MO 312217! [sheet # 2/2]).
Rudgea chiriquiensis= Dwyer, Ann. Missouri Bot. Gard. 67(2): 476, 1980b. ≡ Coussarea chiriquiensis (Dwyer) C. M. Taylor, Fieldiana, Bot., n.s. 33: 113, 1993.—**Type**: PANAMA. Chiriquí: Cerro Colorado, along road above San Félix, 29 km above bridge over Río San Félix, 7.9 km above turnoff to Escopeta, 1500 m, 14 Jul 1976, *T. B. Croat 37071* (lectotype, designated by Taylor (1995): MO barcode MO-312257!; isolectotype: PMA barcode 1163! ex MO 2389189).

#### Nomenclatural remarks.

*Rudgea horquetensis* Dwyer & Hayden was accepted as a species of *Rudgea* by most authors including Lorence (1999) and Correa et al. (2004). Lorence and Taylor (2012) were the first to exclude it from *Rudgea*, but did not suggest any further placement. Based on morphological characters, the species clearly belongs to the nocturnally flowering species group of *Palicourea* (Taylor et al. 2010) and is here treated as conspecific with *Psychotria* or *Palicourea nebulosa*. *Rudgea horquetensis* is the oldest available name for the taxon and a respective new combination is proposed here.

#### Typification.

The protologue of *Coussarea nebulosa* states that the holotype is located at MO. At the time of publication two sheets of the type collection have been accessioned at MO, making the holotype designation ambiguous. In 1975, Dwyer annotated both sheets as *Coussarea nebulosa*, but did not specify what sheet he intended to be the holotype. Hence, both specimens represent syntypes (ICN, Art. 40.2 & Note 1; see also McNeill 2014). Later, the specimens were annotated as sheet “1” and “2 of 2”, respectively. Sheet 1 was also annotated as holotype by C. M. Taylor in 1988.

Taylor (1995) cited the above-mentioned sheets as “holotype, MO 2162999; isotype, MO 4043108” which has to be considered a valid (though indirect) lectotypification according to the ICN (Art. 7.10, 9.9, 9.23; see also McNeill 2014). Later, Lorence (1999) cited the specimens as “Holotype MO 2162995; Isotype MO 4043108”. The last digit of the numbering stamp on the corresponding sheet is hardly legible as a “9”, possibly explaining the error in citation. In the last revision of the group (Taylor et al. 2010), the erroneous type citation of Lorence (1999) was repeated. In a repatriation project in 2001 (C. M. Taylor, pers. comm.), the lectotype (specimen 1) was deaccessioned and distributed to PMA. The sheet still bears a respective MO accession number stamp.

A similar case of indirect lectotypification is found in *Rudgea chiriquiensis.* The species was described with reference to two collections at that time housed at MO, one of which was later distributed to PMA. Likewise, lectotypification (of the MO sheet) was achieved by Taylor (1995).

#### Distribution.

*Palicourea horquetensis* is only known from few sites in Costa Rica and Panama.

### Palicourea tonduzii

Taxon classificationPlantaeGentianalesRubiaceae

(K. Krause) A. C. Berger comb. nov.

urn:lsid:ipni.org:names:77162934-1

Cephaelis tonduziiBasionym. K. Krause, Bot. Jahrb. Syst. 54(3, Beibl. 119): 45–46, 1916. Non Psychotria tonduzii Standl., J. Wash. Acad. Sci. 15(13): 287, 1925b.—**Type**: COSTA RICA. Cartago: Tuis, 650 m, Nov 1897, *A. Tonduz 11461* (lectotype, designated here: fragm. F barcode V0068631F! ex B; syntype, or possibly holotype: B † [photo: F neg. BN-773!]).
Cephaelis discolor= Pol., Linnaea 41(5–6): 572–573, 1877. ≡ Uragoga angosturensis Kuntze, Revis. Gen. Pl. 2: 954, 1891, nom. nov., non Uragoga discolor (Benth.) Kuntze, Revis. Gen. Pl. 2: 960, 1891. Non Psychotria discolor (Griseb.) Rolfe, Bull. Misc. Inform. Kew 1893: 258, 1893, nec Palicourea discolor K. Krause, Bot. Jahrb. Syst. 54(3, Beibl. 119): 40–41, 1916.—**Type**: COSTA RICA. Cartago: Angostura, Nov 1875, *H. Polakowsky 384* (lectotype, designated here: fragm. F barcode V0068625F! ex B; syntype, or possibly holotype: B † [photo: F neg. BN-722!]).
Evea guapilensis= Standl., J. Wash. Acad. Sci. 15(5): 104–105, 1925a. ≡ Cephaelis guapilensis (Standl.) Standl., Publ. Field Mus. Nat. Hist., Bot. Ser. 4(8): 295, 1929. ≡ Psychotria guapilensis (Standl.) Hammel, Selbyana 12: 139, 1991. ≡ Palicourea guapilensis (Standl.) Borhidi, Acta Bot. Hung. 59(1–2): 17, 2017a.—**Type**: COSTA RICA. Limón: Vicinity of Guápiles, 300–500 m, 12–13 Mar 1924, *P. C. Standley 37025* (holotype: US barcode 00129829!).
Evea nana= Standl., J. Wash. Acad. Sci. 15(5): 105, 1925a. ≡ Cephaelis nana (Standl.) Standl., J. Wash. Acad. Sci. 17(7): 171, 1927. **Type**: PANAMA. Colón: Hills N of Frijoles, 19 Dec 1923, *P. C. Standley 27550* (holotype: US barcode 1153871!).
Cephaelis nicaraguensis= Standl., Trop. Woods 16: 46, 1928.—**Type**: NICARAGUA. Atlántico Norte: Puerto Cabezas [Bragman’s Bluff], bank of Kukalaya River, 60 m, 08 Dec 1927, *F. C. Englesing 58* (holotype: F barcode V0068627F!; isotype: fragm. G barcode G00300772! ex F).

#### Nomenclatural remarks.

The earliest published name for the species is *Cephaelis discolor* but combinations under *Psychotria* and *Palicourea* are preoccupied by *Psychotria discolor* (Griseb.) Rolfe and *Palicourea discolor* K. Krause. The next available name *Cephaelis tonduzii* K. Krause cannot be used in *Psychotria* because of the earlier *Psychotria tonduzii* Standl. Hence, the next name *Evea guapilensis* Standl. was adopted and the species became known as *Psychotria guapilensis* (Standl.) Hammel. Recently, Borhidi (2017a) transferred the species to *Palicourea* and proposed the name *Palicourea guapilensis* (Standl.) Borhidi.

However, he overlooked that the earlier published name *Cephaelis tonduzii* K. Krause is still available under *Palicourea* and ought to have been adopted. The corresponding new combination is provided here. It is fortunate that the correct name for this showy species honors Swiss botanist Adolphe Tonduz (1862–1921), a long-term employee at the Museo Nacional de Costa Rica (CR) and one of the most prolific plant collectors in the country.

#### Typification.

*Cephaelis tonduzii* was described with reference to an entire gathering by Tonduz. Krause did not cite a particular herbarium specimen and all possible duplicates are therefore syntypes (ICN, Art. 40.2 & Note 1; see also McNeill 2014). In addition, he was based at B and his working herbarium was destroyed during World War II. Collections made by Tonduz are widely distributed and more or less complete sets are found in CR and US (Stafleu and Cowan 1976–1988). Nevertheless, the only known original material of *Cephaelis tonduzii* is a fragment at F.

The fragment originates from the type at B, as shown by information given on the label. It consists of a capsule with a leaf and part of an inflorescence, which is mounted together with a photograph (“Berlin negative”) of the original B specimen. The removed leaf is clearly recognizable on the photograph providing a definite link between both. Although not the best choice, the fragment at F appears to be the only extant original material and is here designated as lectotype.

A similar situation is found in *Cephaelis discolor*, which was based on a gathering of H. Polakowsky. Likewise, the only known original material is a fragment at F that originated from B. It consists of a leaf, some bracts and fruits in a capsule, which is mounted together with a photography of the B specimen, likewise destroyed. The fragment at F is here designated as lectotype.

#### Distribution.

*Palicourea tonduzii* is known from Nicaragua, Costa Rica, Panama and Ecuador (Taylor 2014).

### Palicourea longiinvolucrata

Taxon classificationPlantaeGentianalesRubiaceae

A. C. Berger nom. nov.

urn:lsid:ipni.org:names:77162935-1

Psychotria hispidulaBasionym/replaced synonym. Standl. ex Steyerm., Acta Biol. Venez. 4(1): 97–98, 1964. ≡ Palicourea hispidula (Standl. ex Steyerm.) Borhidi, Acta Bot. Hung. 59(1–2):17, 2017a, nom. illeg. hom., non Palicourea hispidula Standl., Publ. Field Mus. Nat. Hist., Bot. Ser. 11(5): 227, 1936.—**Type**: COLOMBIA. Valle del Cauca: Río Calima, La Trojita, 5–50 m, 19 Feb–10 Mar 1944, *J. Cuatrecasas 16359* (holotype: F barcode V0070450F!; isotypes: BC 623883!, U barcode 0006197!, US barcode 00138790!, VEN n.v.).
Psychotria involucrata– sensu Standley (1938, p.p.), non Sw., nom. superfl.
Psychotria hoffmannseggiana– sensu Burger & Taylor (1993, p.p.), non (Roem. & Schult.) Müll. Arg.

#### Nomenclatural remarks.

**Figure 1.**
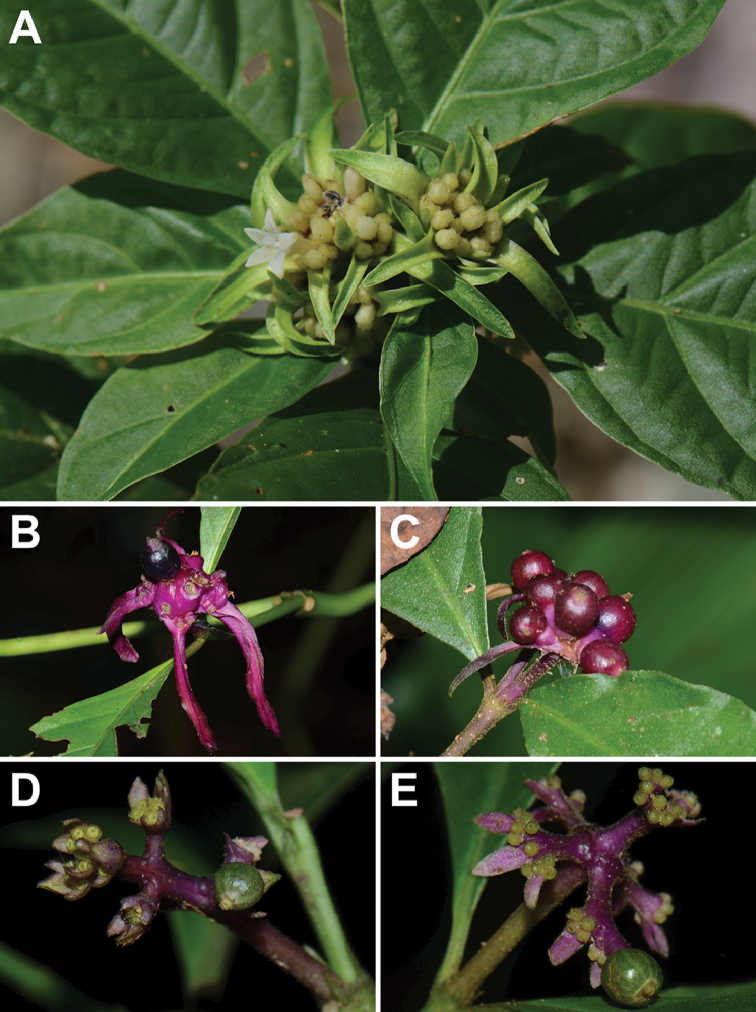
*Palicourea longiinvolucrata* and related species, note diagnostic differences in bract arrangement and length. *Palicourea longiinvolucrata*, inflorescence (**A** *Berger 1418*) and infructescence (**B** *Berger 1633*); *Palicourea hoffmannseggiana*, infructescence (**C** unvouchered); *Palicourea gracilenta*, infructescence (**D** *Berger 2055*); *Palicourea winkleri*, infructescence (**E** *Berger 1737*). All photos by the author.

Borhidi (2017a) attempted to transfer this species to *Palicourea*, but overlooked the preexisting *Palicourea hispidula* Standl. This renders his name an illegitimate later homonym (ICN, Art. 53.1). Here, the new name *Palicourea longiinvolucrata* is proposed that alludes to the long involucral (15–20 mm) and floral bracts (6–15 mm) that help differentiate this species from the closely related *Palicourea hoffmannseggiana* (Roem. & Schult.) Borhidi (3.5–15 mm, 0.5–2 mm). Two further species, *Palicourea gracilenta* (Müll. Arg.) Delprete & J. H. Kirkbr. (3–7 mm, 1.5–5 mm) and *Palicourea winkleri* Borhidi (both 1–9 mm), belong to the same species group (e.g., Taylor 2004, 2014), but have less-congested inflorescences (Figure 1).

#### Distribution.

*Palicourea longiinvolucrata* is known from Belize to Bolivia, from Venezuela and from Brazil (Taylor 2014).

## Supplementary Material

XML Treatment for Palicourea horquetensis

XML Treatment for Palicourea tonduzii

XML Treatment for Palicourea longiinvolucrata
